# Impact of pore anisotropy on the thermal conductivity of porous Si nanowires

**DOI:** 10.1038/s41598-018-30223-0

**Published:** 2018-08-24

**Authors:** P. Ferrando-Villalba, L. D’Ortenzi, G. G. Dalkiranis, E. Cara, A. F. Lopeandia, Ll. Abad, R. Rurali, X. Cartoixà, N. De Leo, Z. Saghi, M. Jacob, N. Gambacorti, L. Boarino, J. Rodríguez-Viejo

**Affiliations:** 1 Grup de Nanomaterials i Microsistemes, Departament de Física, Universitat Autònoma de Barcelona, 08193 Bellaterra, Spain; 2 Nanofacility Piemonte INRiM, Nanoscience & Materials Division, Istituto Nazionale di Ricerca Metrologica, Strada delle Cacce 91, 10135 Torino, Italy; 3 IMB-CNM-CSIC, Campus Bellaterra, 08193 Bellaterra, Spain; 4 Institut de Ciència de Materials de Barcelona (ICMAB–CSIC), Campus de Bellaterra, 08193 Bellaterra, Spain; 5 Departament d’Enginyeria Electrònica, Universitat Autònoma de Barcelona, 08193 Bellaterra, Spain; 6 University of Grenoble Alpes, Grenoble F-38000, France; CEA, LETI, MINATEC Campus, Grenoble, F-38054 France

## Abstract

Porous materials display enhanced scattering mechanisms that greatly influence their transport properties. Metal-assisted chemical etching (MACE) enables fabrication of porous silicon nanowires starting from a doped Si wafer by using a metal template that catalyzes the etching process. Here, we report on the low thermal conductivity (*κ*) of individual porous Si nanowires (NWs) prepared from MACE, with values as low as 0.87 W·m^−1^·K^−1^ for 90 nm diameter wires with 35–40% porosity. Despite the strong suppression of long mean free path phonons in porous materials, we find a linear correlation of *κ* with the NW diameter. We ascribe this dependence to the anisotropic porous structure that arises during chemical etching and modifies the phonon percolation pathway in the center and outer regions of the nanowire. The inner microstructure of the NWs is visualized by means of electron tomography. In addition, we have used molecular dynamics simulations to provide guidance for how a porosity gradient influences phonon transport along the axis of the NW. Our findings are important towards the rational design of porous materials with tailored thermal and electronic properties for improved thermoelectric devices.

## Introduction

The reduction in the dimensionality of solid materials at the nanoscale sharply reduces their thermal conductivity by adding new scattering terms that shorten the phonon relaxation time. The impact of boundary scattering, surface roughness and/or composition has been demonstrated in many semiconductors^1–13^. Another strategy to reduce the thermal conductivity is to drill the sample with holes to enhance phonon interface scattering^14,15^. Porous materials typically have inner irregular holes with sizes and separations of a few nanometers that vastly increase the surface-to-volume ratio. These holes are potentially interesting for a variety of applications including thermal insulation, sensors, or energy-efficient thermoelectric devices. For silicon materials, porous Si can be readily etched from the bulk and integrated into silicon microfabricated devices. Generally, porous Si is produced by electrochemical etching with an aqueous solution of HF^16,17^, although an ethanoic solution removes H_2_ bubbles more efficiently. The resulting chemical reaction in this method is the fluorination of Si atoms, which, combined with the instability of the surface charges, induces a pore nucleation into the Si that facilitates the penetration of the solution into the pores. Interestingly, porous Si produced by wet etching consists of an interconnected network of very thin single-crystalline Si regions, the microstructure of which strongly depends on the doping level of the initial substrate and on the preparation method^18^. The electrochemical etching process applied to highly doped silicon is known to produce columnar pores that are preferentially oriented in the out-of-plane direction^19,20^. This microstructure greatly impacts the macroscopic properties of transport, as these properties exhibit anisotropy depending on the direction of the measurement with respect to the orientation of the pores. In particular, the in-plane thermal conductivity, where phonon transport is perpendicular to the average pore orientation, is 20–100 times smaller than the cross-plane conductivity where heat flows parallel to the pores^21^. Thermal conductivity values in porous bulk Si range from 0.2 to 4 W·m^−1^·K^−1^ at 300 K^16,22–27^. These values correspond to an effective medium considering the material to be homogeneous. Hydrodynamic-like approaches have reproduced the very low thermal conductivity of these porous structures, and an important relationship between $\kappa$ and the pore diameter has been found^28,29^. Cross-plane and in-plane electrical measurements show a similar anisotropy in the conductivity of mesoporous silicon (100), exhibiting a ratio of five orders of magnitude for the electrical conductance between two electrodes in a sandwich configuration and two guarded planar contacts on the top surface of the mesoporous silicon. This electrical anisotropy of p+ mesoporous silicon revealed a typical threshold in the transversal direction with respect to the columnar structure, providing evidence of a Coulomb blockade at room temperature in this system^30^.

More recently, part of the knowledge gained by studying the electrochemical formation of porous silicon has been applied to the production of silicon nanowires using metal-assisted chemical etching (MACE), an electroless method originally proposed by Li & Bohn^31–33^. In this method, the substrate is covered with a patterned metallic thin film that acts as a catalyst for the etching. The metal, typically gold or silver, catalyzes the oxidation reaction of the Si underneath by H_2_O_2_, while HF etches SiO_2_ away. In this way, the metallic layer sinks into the Si, extruding silicon structures with shapes strongly dependent on the initial metal patterning. By combining different lithography approaches, 3D structures can be obtained with aspect ratios that are in some cases competitive with deep reactive ion etching^34,35^. In the literature, few papers report thermal conductivity measurements in porous Si nanowires that are prepared by MACE^33,36–40^, most of them performed on large arrays of Si nanowires (NWs), with values around 1.7 W·m^−1^·K^−1^ that approach the amorphous limit. The increase in porosity was found to decrease the thermal conductivity, enhancing the Seebeck coefficient and boosting $ZT$ to 0.4^33^. As in the case of Coulomb blockade in electrical transport, the smaller the points of contact in the silicon skeleton, the higher the thermoelectric figure of merit, mainly due to the increase in the surface-to-volume ratio of the wire. Measurements on single nanowires enable accurate process-structure-property relations that take into account the effect of the nanostructure of the nanowires on the measured property. With respect to thermal transport in individual NWs the use of suspended platforms is crucial to keep thermal losses as low as possible. Using these microfabricated devices the thermal conductance of samples with very low thermal conductance has been successfully measured^41,42^. Low values of *κ* have already been obtained in 50 nm diameter Si NWs with rough surfaces produced by electroless etching^43,44^. In contrast, smooth wires with the same diameter only show a six-fold reduction^1^. Crystalline Si nanotubes with walls in the range of 5 nm exhibit sub-amorphous thermal conductivity^5^. Concerning porous silicon a very recent paper reports the thermal conductivity measurement of single porous Si NWs prepared by MACE with conductivity values ranging from 0.33-to-1 Wm^−1^K^−1^ in a porosity range of 30–50%^45^.

Here we report the thermal conductivity of single porous Si NWs ranging in diameter from 90 to 160 nm. The low values of the effective thermal conductivity, between 0.8 and 1.5 W·m^−1^·K^−1^, confirm the enhanced scattering impact of porosity, which should then suppress any size dependence as a result of the subordinated role of boundary scattering. Unexpectedly, we do observe an increase in the thermal conductivity with the diameter of the nanowires. 3D morphological analysis by electron tomography demonstrate the presence of longitudinal pores along the NW axis in the core region and the existence of lateral pore branching at the outer edge with pores preferentially oriented towards the surface of the wire. The impact of pore anisotropy on the measured thermal conductivity is further analyzed by molecular dynamics simulations using a simple core/shell model.

## Results and Discussion

### Thermal conductivity measurements

**Figure 1:**
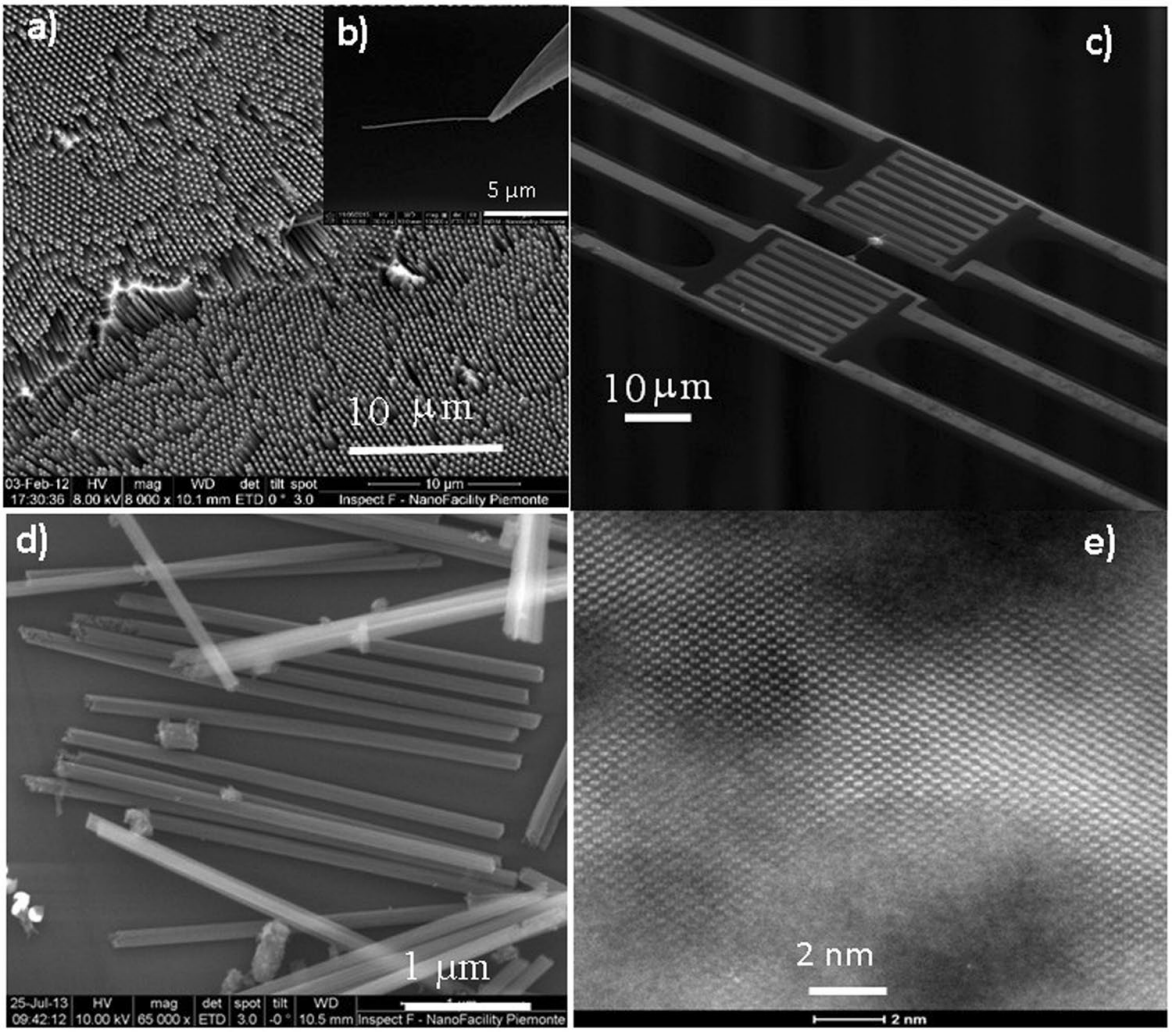
(**a**) Nanowire forest created with MACE. (**b**) The nanomanipulator grabbing a wire. (**c**) Suspended structure after hanging the wire between the platforms for heating and sensing. (**d**) Micrograph of porous Si NWs. (**e**) HRTEM image of a nanowire showing the existence of crystalline regions.

The NWs were top-down fabricated using self-assembly of polystyrene nanospheres and MACE, which allows the production of very long nanowires with the desired diameter and porosity by tuning the wafer doping and the etchant concentration (more details can be found in SI). The NWs (Fig. 1a) were transferred with a nanomanipulator (b, inset of Fig. 1a) to the microdevice and bridged between two suspended platforms for the thermal conductivity measurements (Fig. 1c). Figure 1d obtained with a field emission gun scanning electron microscope (FEG-SEM) confirms the diameter of the NWs is constant over their length, while the High Resolution Transmission Electron Microscopy (HRTEM) picture in Fig. 1e shows the presence of crystalline regions around the pores highlighting the crystalline skeleton of the NW with sizes around 5–10 nm.

**Figure 2:**
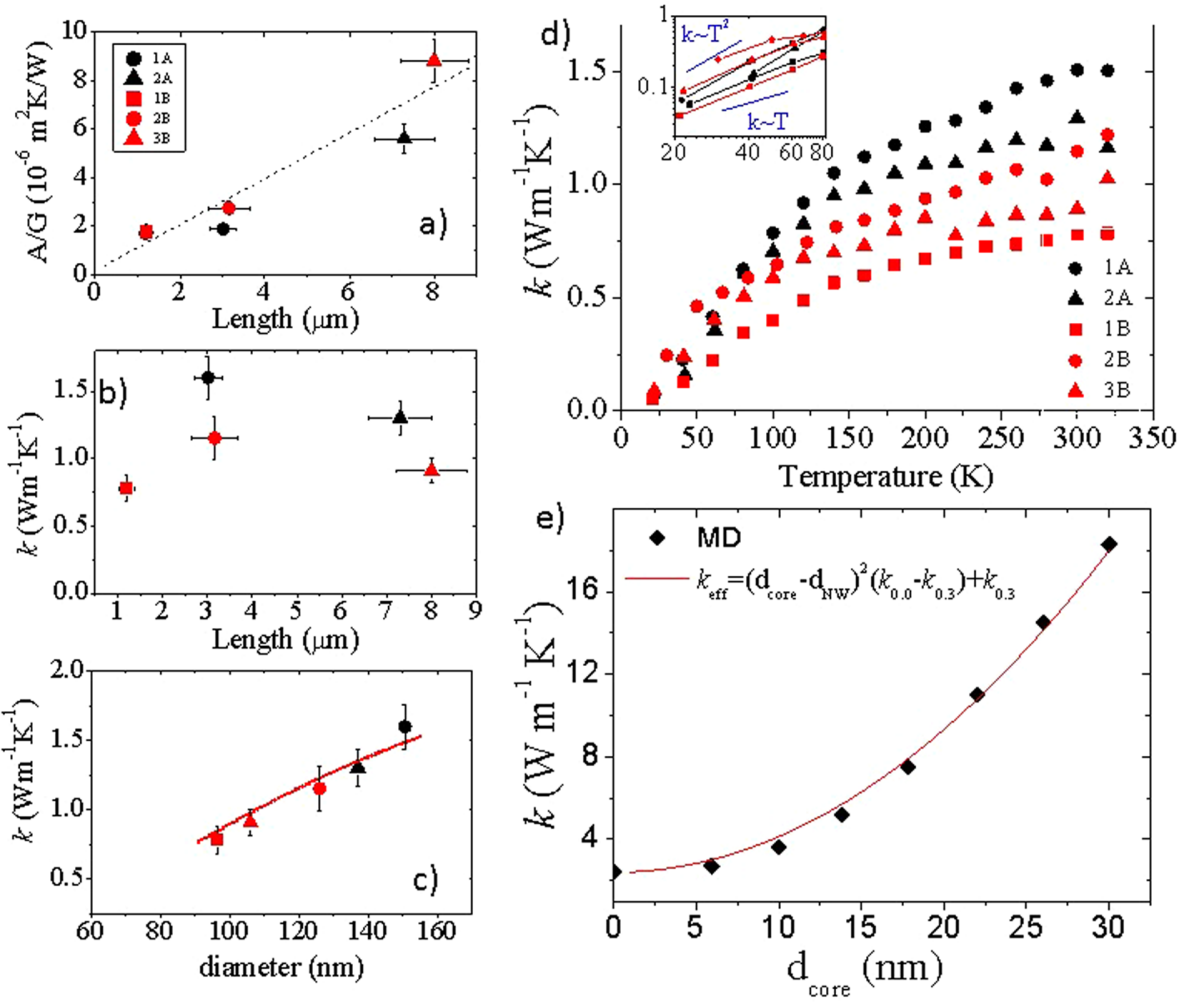
(**a**) Thermal resistance multiplied by the area as a function of length. The legend indicates the reference number of the suspended structure and the batch of the NW. (**b**) Thermal conductivity as a function of length and (**c**) diameter. (**d**) Thermal conductivity as a function of temperature. Inset: Thermal conductivity in the low-T region plotted logarithmically. (**e**) Thermal conductivity of core-shell NW calculated by molecular dynamics as a function of the core diameter (diamonds). The continuous red lines in (**c**) and (**e**) are the effective thermal conductivities calculated as ${{\kappa }}_{{eff}}={({{d}}_{{core}}/{{d}}_{{NW}})}^{2}({{\kappa }}_{{c}}-{{\kappa }}_{{shell}})+{{\kappa }}_{{shell}}$, assuming that the overall conductivity results from two conductive channels in parallel: a core with conductivity ${{\kappa }}_{{c}}$ and diameter ${{d}}_{{core}}$ and a shell with conductivity ${{\kappa }}_{{shell}}$ and thickness ${{d}}_{{NW}}-{{d}}_{{core}}$, where ${{d}}_{{NW}}$ is the diameter of the NW. The dashed line in (**a**) is a guide to the eye.

Thermal conductance measurements were performed using a differential configuration by subtracting the base conductance of the empty structure (which comprises conduction through the substrate) from the measured wire conductance, yielding a very low measurement error similar to the measurement noise, around 20 pW/K. The parasitic background thermal contribution is small, around 0.61 nW/K, but it can significantly influence the final value, i.e., by up to 30% for wires with the lowest conductance. Therefore, for each individual microdevice, we subtract the residual thermal background to obtain accurate measurements of the thermal conductance. More details are given in the Supplementary Information (SI). Subsequently, the NWs were anchored to the platforms in a focus ion beam (FIB) instrument (FEI Quanta3D ESEM FEG Dual beam FIB-SEM) equipped with Kleindiek Nanoteknik nanomanipulators and by means of ion-induced Pt deposition. We verified that this process applied at the contacts does not adversely affect the thermal conductance in these low conductivity nanowires. The thermal contact resistance, R_th_, was evaluated by measuring the thermal conductance of NWs of different lengths. Since phonon transport is highly diffusive in these porous materials, the thermal conductance should have a linear dependence with the length *l* of the wire, provided that R_th_ is similar for all wires. To account for the different diameters of the nanowires, we normalized the thermal resistance (*1/G*) by the section of the nanowire (*A*), and thus we plot (*A/G*) vs. *l* in Fig. 2a. The intersection with the y-axis provides an estimate of R_th_, which in this case is lower than 10^−6^ m^2^ KW^−1^. This represents less than 15% of the total thermal resistance for the longest nanowire. All measured nanowires exhibit a drastic reduction in the effective thermal conductivity, with values ranging from 0.8 to 1.5 W·m^−1^·K^−1^ as shown in Fig. 2b. In addition, Fig. 2b highlights that there is not a linear trend of the conductivity with the length of the wire, meaning that indeed transport in the porous material is mostly diffusive in nature. However, variations exist and the wires with an intermediate length have higher thermal conductivity values than the others. Table 1 in the experimental section shows that such wires have the highest diameter. In fact, a plot of the thermal conductivity as a function of wire diameter evidences a hidden correlation with the diameter (Fig. 2c). Irrespective of the batch, larger diameter nanowires tend to be more conductive. Previous work on crystalline Si NWs ranging from 30 to 120 nm in diameter has showed that heat carriers are strongly scattered by the wire surface; therefore, there is a strong dependence of the conductivity on the diameter of the wire. At a high surface roughness, the thermal conductivity can even be reduced by two orders of magnitude compared to bulk Si^44,46^. The thermal conductivity of porous solids is greatly reduced due to the suppression of large wavelength phonons. Then, we may wonder if the conductivity of wires embedded with large porosities ($\sim 40 \%$) and small crystalline regions (5–10 nm) should exhibit any dependence at all with the diameter of the wire, since phonon scattering at the pore boundaries should dominate over the scattering with the external surface. A recent simulation study^47^ showed, in fact, that the impact of boundary scattering on the thermal conductivity decreases drastically for nanowires with 30% porosity and diameters greater than 35 nm. Assuming that pore scattering dominates heat transport, the behavior shown in Fig. 2c could be rationalized by considering the existence of porosity inhomogeneities, either in the form of porosity gradients along the radius of the nanowire, as the core may be less porous on average than the shell, or by a change in the microstructure/orientation of the pores from the surface toward the center of the wire. This point is further discussed below.

**Table 1:** Length and diameter of the Si nanowires measured in this work.

Sensor (sample)	Recipe (HF:H_2_O_2_:H_2_O)	Length (μm)	Diameter (nm)
1A	3:1:1 (A)	3.0 ± 0.3	151 ± 5
2A	3:1:1 (A)	7.3 ± 0.7	137 ± 5
1B	30:1:30 (B)	1.2 ± 0.2	96 ± 5
2B	30:1:30 (B)	3.2 ± 0.5	126 ± 5
3B	30:1:30 (B)	8.0 ± 0.8	106 ± 5

The thermal conductivity variation with temperature (Fig. 2d) is dramatically different from that of bulk Si, showing a modest increase and even a saturation of the conductivity in the mid-temperature range (150–300 K). This is very similar to amorphous and nanocrystalline solids^5,48^ and has also been previously reported in rough Si nanowires and porous materials^16,44^. The enhanced phonon boundary scattering in these materials and its dominance over the Umklapp scattering explains the difference with crystalline silicon above 100 K. In the low temperature region ($T < 80\,{\rm{K}}$), the variation of the thermal conductivity is also very different from that in crystalline bulk solids. The latter, in agreement with Debye theory, show a ${T}^{3}$ dependence of $\kappa$, while porous nanowires have lower temperature exponents between 1 and 2 (inset of Fig. 2d). This is compatible with the findings in other nanostructures near the confinement regime^1,49–51^, which suggests the frequency dependence of specularity in boundary and pore phonon scattering^52^.

### Molecular dynamics simulations

The thermal conductivity values shown in Fig. 2 are effective values, as we assumed that the wires are homogeneous. We can use a simple yet effective approach, based on the Eucken model^53,54^, to estimating the thermal conductivity of bulk silicon, *κ*_*Si*_. In the presence of nanometer sized pores, an extra term accounting for the interpore distance must be added to the Eucken expression^47,55^:

1 $${\kappa }_{Si}=\frac{{\kappa }_{eff}}{F}$$

2 $$F=\frac{1-p}{1+\frac{p}{2}+\frac{3{\Lambda }_{{\rm{bulk}}}}{2{d}_{p}}p}$$

where $p$ is the porosity of the wire, ${d}_{p}$ is the average pore diameter and ${\Lambda }_{{\rm{bulk}}}$ is the average phonon mean free path in the nonporous material. Applying this factor with the measured porosity of the wires ($\sim 40 \%$) on the longest wire and taking a representative value of ${\Lambda }_{\mathrm{bulk},\mathrm{Si}}\sim 70$ nm and using ${d}_{p}\sim 10\,nm$, we get ${\kappa }_{Si}\sim 16.7$ W·m^−1^·K^−1^, which is around one-ninth of the bulk silicon thermal conductivity. This value provides an inaccurate estimate since it is based on a homogeneous distribution of pores within the nanowire, which we know does not represent the true porous distribution. Also, note that the NW diameters we have are much larger than the interpore distance, around 5–10 nm, and thus, in principle, surface scattering should be much less frequent than the inner pore boundary scattering, leading to an independence of *κ* on the NW diameter, at odds with our experimental observations. To underpin the mechanism leading to this strong reduction in the thermal conductivity and the dependence of *κ* on the NW diameter, we performed atomistic approach-to-equilibrium molecular dynamics (AEMD) simulations. We considered a Si NW with a diameter of 30 nm and a coaxial structure consisting of a monocrystalline core and a porous shell. The diameter of the core, *d*_*core*_, is varied from 0 to 30 nm and the thickness of the shell is scaled accordingly; the porosity of the shell is 30%. Clearly, this is an oversimplified model of the morphology observed experimentally and described below, but it qualitatively captures the underlying physical mechanisms, i.e., the effect of a radially non-uniform porosity on the thermal conductivity. We also need to restrict to thinner NW than those used in the experiment, yet sufficient to account qualitatively for the basic underlying physics, in order to keep the computational load at a manageable level. The results are shown in Fig. 2e, where we plot κ as a function of the core diameter. The thermal conductivity changes from 18.2 W·m^−1^·K^−1^ for the monocrystalline NW (*d*_*core*_ = 30 nm) to 2.2 W·m^−1^·K^−1^ for the fully porous NW (*d*_*core*_ = 0), thus supporting the dependence of κ on the internal structure of the porosity observed experimentally. The conductivity values may be slightly overestimated due to the use of the Tersoff potential in the AEMD simulations. Interestingly, the calculated values of *κ* in Fig. 2e are nicely approximated by the simplest possible model, in which heat is carried by two parallel transport channels: a core with conductivity *κ*_*core*_ = *κ*_*0*.*0*_ and a shell with *κ*_*shel*l_ = *κ*_*0*.*3*_; *κ*_*0*.*0*_ and *κ*_*0*.*3*_ are the thermal conductivities of a 30 nm monocrystalline NW and porous NW with porosity 30%, respectively.

### Microstructural analysis of the porosity distribution

**Figure 3:**
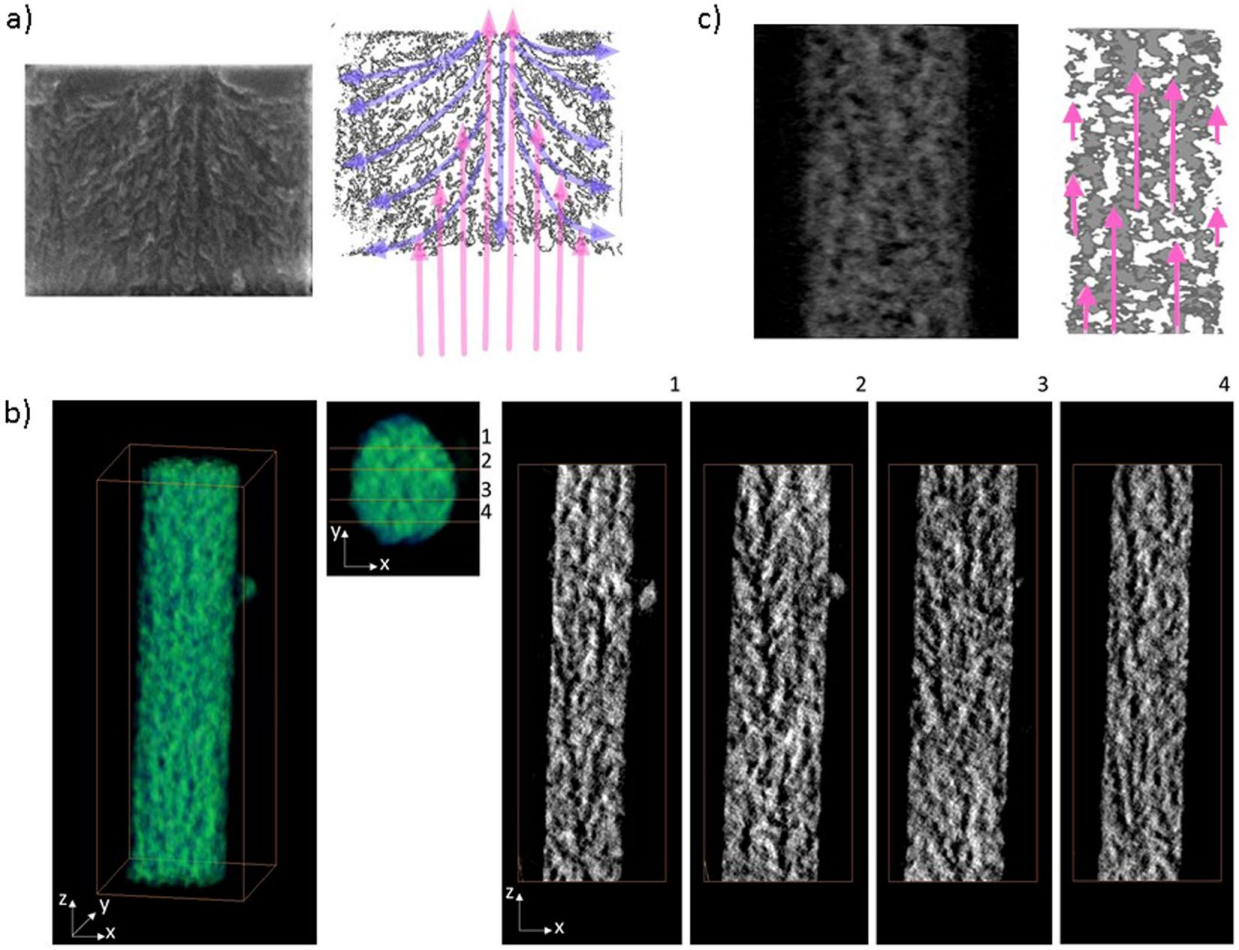
(**a**) Cross-section SEM micrographs of 400–500 nm porous Si NW, showing the development of porosity into Christmas tree–like structures due to the etching mechanism. The violet lines illustrate the propagation of the pores. The pink arrows indicate the direction of heat flow during the thermal measurement (**b**) 3D reconstruction of a selected NW (96 nm in diameter) by HAADF-STEM tomography: The left hand side image is the volume rendering of the reconstruction. Pictures 1–4 represent x-z cross-sections at different positions through the NW. Darker zones represent the pores. (**c**) Detailed view of a section of the NW showing the porosity (darker regions) and at the right a digitally processed image to highlight the percolation lengths for heat flow as schematically indicated with the pink arrows.

To obtain a more realistic picture of the variation of the thermal conductivity with the diameter of the nanowires, we conducted a detailed microstructural analysis of the porous structure of the Si NWs both with SEM cross sections (Fig. 3a,b) and electron tomography. We provide evidence that the specific etching procedure yields a porosity distribution that may affect the thermal conductivity of the nanowires. The Si NWs produced by MACE from highly doped substrates are porous, because the free holes in the material not only contribute to localized electropolishing at the metal-silicon interface, but also are available to reach the silicon nanowires walls with the help of the electric fields induced by the redox potential between metal and silicon. For large porous NWs of diameter around 400–500 nm there is an obvious asymmetry in the porosity distribution with a Christmas tree-like structure induced by the etching mechanism (Figs 3a and S2). In the small diameter NWs measured in this work, the asymmetry of the pores is not evident in the cross-section SEM images of the cleaved NWs (Fig. S2d), but can be visualized in cross-sectional (tomographic) images of the 3D reconstructed NW (Fig. 3b) that show the existence of a thunder-like morphology with longitudinal pores along the wire axis in the core region and lateral pore branching.

The microstructure of the NW is composed of a central region with longitudinal pores and a silicon skeleton, which favors electrical and thermal transport along the wire length because of the large percolation pathways. On the contrary at the outer shell the presence of laterally oriented pores that reach the wire walls drastically decrease the percolation length of the thermal carriers along the wire axis. This behavior is schematically represented by the length of the pink arrows in Fig. 3b. In this region, room temperature Coulomb blockade and low thermal conductivity are expected. Similar to that previously observed for bulk silicon^21^, pore anisotropy in the nanowires should strongly affect their thermal conductivity. In a simplified approach we model the pore anisotropy as a core-shell representation, with the two regions having different orientations of the pores with respect to the heat flow. In this model, heat is carried by two parallel transport channels with very little interaction between them: a core with conductivity *κ*_*core*_ and a shell with conductivity *κ*_*shell*_. Based on the anisotropic pore distribution of Fig. 3, the outer shell consists of small crystalline regions separated by pores that are oriented towards the outer surface and nearly perpendicular to the heat flow. In this region, the heat transport is severely limited by phonon scattering with the pores. At the inner core, the pores and the interconnected silicon networks are, on average, distributed along the axis of the wire, and transport proceeds within the crystalline material, which is a few nm in diameter. Consequently, *κ*_*core*_ > *κ*_*shell*_. The core-shell model also captures the trend displayed in the experimental data, as shown by the continuous line in Fig. 2c. In this case, the equation used to fit the data was

3 $${\kappa }_{eff}={(\frac{({d}_{NW}-62)}{{d}_{NW}})}^{2}\cdot ({\kappa }_{core}-{\kappa }_{shell})+{\kappa }_{shell}$$

where *κ*_*core*_ = 3.4 Wm^−1^ K^−1^, *κ*_*shell*_ = 0.45 Wm^−1^ K^−1^, the inner region being 7.5 times more conductive that the shell region, and *d*_*NW*_ is the measured diameter of the nanowire. To fit the experimental data with Eq. (3), we assume that the diameter of the inner core varies as d_core_ = d_NW_ − 62 nm, that is, from 30 to 90 nm. Since the larger diameter NWs have a larger core region they display a higher thermal conductivity with respect to smaller NWs in which thermal transport is more affected by the porous structure of the shell region. The thermal conductivity values measured on the porous Si NWs of this work are lower than those previously obtained for rough Si nanowires of comparable diameter.

## Conclusions

Single-crystalline porous Si NWs with diameters ranging from 90–150 nm were fabricated by MACE and nanosphere self-assembly, and their thermal conductivity evaluated using suspended platforms. Thermal conductivity values as low as 0.87 Wm^−1^ K^−1^ were measured with a significant dependence with the diameter of the NW. Electron tomography revealed a 3D porosity distribution with pores oriented on average parallel to the NW axis in the core region and up to 60° with respect to the NW axis at the shell region. It was found that pore anisotropy has a significant influence on the thermal conductance evaluated along the axis of the nanowire. The anisotropic pore distribution could be used to further tune electrical and thermal conductivities towards improved electronic and thermoelectric devices. MD simulations showed that a simple core-shell model with two effective thermal conductivities is a useful approach to explain the measured data. Due to their low thermal conductivity, in general, porous wires and porous Si are promising materials for improved thermoelectric generation and sensing.

## Methods

### Nanowire fabrication

The porous Si nanowires were produced using metal-assisted chemical etching (MACE). The main fabrication steps are summarized below and are further detailed in the SI (Figs S1 and S2). The process starts with colloidal lithography, consisting of small polystyrene nanospheres, synthesized by emulsion polymerization, that are dispersed on the wafer. The nanospheres are in contact with each other, so they must be reduced in order to produce separated holes in the gold thin film. This is achieved with an Ar + O_2_ plasma that selectively etches the organic spheres, effectively reducing their radius. Subsequently, a 20 nm thick Au layer is deposited by means of e-beam evaporation. Then, the nanospheres are removed by sonicating the wafer in DI water. The result is a holey Au mask that will act as a template for the wires formation. The nanowires are defined by immersing the wafer into wet etching solutions composed of HF, H_2_O_2_, and H_2_O with proportions 30:1:30 and 3:1:1. However, the final porosity of the nanowire is largely dominated by parameters such as substrate doping, metallization condition, geometry and the defectivity of the anti-dot gold mask. Using this approach, we produced very long nanowires (~30 microns) as shown in Fig. 1a. In this work, (100) B-doped p^+^-Si wafers with a resistivity of 0.008–0.012 Ohm·cm were used.

### Device fabrication and manipulation

The microsensors consist of two long (400 μm) bridges that each support a Pt element that works as heater and sensing elements (see Fig. 1c). The separation between bridges ranges from 1 to 10 μm for different devices, which allows hanging wires with lengths in the same range. The wafers with the nanowires on top are introduced into a SEM/FIB instrument together with the suspended structures (described elsewhere^3^) where the wires are to be attached. Using a nanomanipulator with a sharp needle (radius ~ 200 nm), one wire is bonded through ion-induced deposited Pt (Fig. 1b). Once this is achieved, the wire is cut several microns below using the FIB. The detached wire is then placed on the sensing platform, where it is bonded using Pt again, and then it is detached from the needle by cutting it again with the Ga^+^ FIB. As stated previously^3^, Ga^+^ ions can introduce disorder into the Si lattice near the cutting area. The use of an ion beam to deposit Pt will probably amorphize the region under the contact and will produce some nearby defects. Also, electrical measurements on a similar Si NW confirm the efficiency of this amorphization in terms of electrical conductivity. However, since the porous wires exhibit very low thermal conductance, we expect the enhanced thermal resistance to have a small influence on the derived thermal conductivity data.

### Thermal conductivity measurements

We measured the thermal conductivity of the nanowires presented in this paper using the suspended structures technique. By heating one of the bridges of the structure and measuring the temperature rise on both supporting platforms, the thermal conductance of the nanowire bridging both platforms is derived^56^. The thermal conductivity was estimated using the apparent cross-sectional area of the NWs as deduced from their diameter. The measurements were performed inside a He cryostat at temperatures ranging from 20 K to 320 K using a commercial temperature controller, and at pressures below 10^−5^ mbar to avoid convection losses. A double radiation shield was used to enhance the accuracy of the R(T) calibration^57^. To handle the background conduction through the substrate, we subtracted the conductance measured in the empty suspended structure. Finally, the additional resistance due to the presence of amorphous Si in the supporting membrane of some wires was subtracted using finite element modeling. More detailed information about the modeling and background conductance can be found in the SI.

### Finite element modeling

Two of the porous nanowires measured were bonded to the Si membrane hanging between both heaters in the suspended structure. In this case, the previous cutting of the membrane and the latter cutting of the wire amorphized the borders of the membrane. In order to subtract the added thermal resistance of such an amorphous membrane from the measured values, these structures were 3D modeled with the software COMSOL. More information about this simulation can be found in the SI.

### Molecular dynamics simulations

We performed fully atomistic simulations using approach-to-equilibrium molecular dynamics (AEMD)^58,59^, in which the transient between a suitably designed non-equilibrium condition to equilibrium carries information about the thermal diffusivity. After a careful equilibration cycle, a step-like temperature profile was set up by velocity rescaling for 200 ps for each half, after which the system was left free to evolve microcanonically for 1 ns. By tracking the evolution of ${\rm{\Delta }}T=\langle {T}_{L}\rangle -\langle {T}_{R}\rangle$ with time, where $\langle {T}_{L}\rangle$ and $\langle {T}_{R}\rangle$ are the average temperatures of the halves of the system initially equilibrated at a hot and a cold temperature, respectively, we fitted the numerical data to the analytical solution of the heat equation. This procedure allowed us to extract the thermal diffusivity, $\bar{\kappa }$, which, in turn, gives the thermal conductivity, $\kappa =\rho {C}_{v}\bar{\kappa }$, where $\rho$ is the density and ${C}_{v}$ is the specific heat capacity. We used the environment-dependent interatomic potential (EDIP)^60^ for the calculation of the energy and forces, and we used a time step of 2 fs throughout all of the simulation protocol. The initial values of ${T}_{L}$ and ${T}_{R}$ were 400 K and 200 K, respectively (notice that previous studies showed that the results do not depend significantly on these values^59^). The full simulation details can be found in Cartoixà *et al*.^47^.

### Structural characterization

Several Si nanowires from the same batches measured in this work were characterized by FE-SEM and TEM, including 3D tomography to obtain a high-resolution map of the embedded porosity of the wires. First, FE-SEM images (Fig. 1c) were taken just after placing the wires on the suspended structures in order to measure the length and diameter of each wire, which enabled the proper calculation of the thermal conductivity. The micrographs were obtained with the secondary electrons in a field emission SEM at 20/30 KV and 100 pA. The length was measured between the deposited Pt spots, but since these are sometimes poorly defined due to Pt diffusion, the value may have an added uncertainty. The diameter was measured at 2 or 3 different spots on the nanowire to account for possible variability along its length. The wire diameter ranged between 96 and 151 nm, while the length ranged between 0.96 and 8 μm. The exact values for each wire are shown in Table 1.

TEM images with magnification up to x950000 were taken from nanowires in each batch to measure the pore size and internal structure (Fig. 1e). They showed very small pores, with sizes about 6–8 nm for the A-type wires and 10–12 nm for the B-type wires. However, it was not possible to estimate the size of the pores from a single transmission image in one direction, since the entire thickness of the wire was being observed. Therefore, previous values were taken as rough estimations. The crystalline phase between pores was clearly observed in all analyzed samples, as shown in Fig. 1e.

High angle annular dark field scanning TEM (HAADF-STEM) tomography was performed on a selected A-type nanowire using a FEI Titan Themis microscope operating at 200 kV. HAADF-STEM images were acquired between −72° and +62° with a 2° increment and a pixel size of 0.53 nm. The tilt series alignment and 3D reconstruction by SIRT algorithm (simultaneous iterative reconstruction technique) were performed using an in-house tomography package. Avizo software was then used for the 3D visualization of the pores inside the nanowire (Fig. 1c). From these data, the porosity can be calculated by subtracting the volume occupied by the vacuum to the total volume of the nanowire, resulting in 37% porosity. Nevertheless, this value has quite high uncertainty due to several factors. First, it depends on how the segmentation is performed, this is, where the limit between material and vacuum is imposed. Second, the differentiation between the surface roughness and internal porosity can be ambiguous.

## Electronic supplementary material

Supplementary Information
