# Author Correction: Impact of pore anisotropy on the thermal conductivity of porous Si nanowires

**DOI:** 10.1038/s41598-018-32320-6

**Published:** 2018-10-04

**Authors:** P. Ferrando-Villalba, L. D’Ortenzi, G. G. Dalkiranis, E. Cara, A. F. Lopeandia, Ll. Abad, R. Rurali, X. Cartoixà, N. De Leo, Z. Saghi, M. Jacob, N. Gambacorti, L. Boarino, J. Rodríguez-Viejo

**Affiliations:** 1 Grup de Nanomaterials i Microsistemes, Departament de Física, Universitat Autònoma de Barcelona, 08193 Bellaterra, Spain; 2 Nanofacility Piemonte INRiM, Nanoscience & Materials Division, Istituto Nazionale di Ricerca Metrologica, Strada delle Cacce 91, 10135 Torino, Italy; 3 IMB-CNM-CSIC, Campus Bellaterra, 08193 Bellaterra, Spain; 4 Institut de Ciència de Materials de Barcelona (ICMAB–CSIC), Campus de Bellaterra, 08193 Bellaterra, Spain; 5 Departament d’Enginyeria Electrònica, Universitat Autònoma de Barcelona, 08193 Bellaterra, Spain; 6 University of Grenoble Alpes, Grenoble F-38000, France; CEA, LETI, MINATEC Campus, Grenoble, F-38054 France

Correction to: *Scientific Reports* 10.1038/s41598-018-30223-0, published online 24 August 2018

In the original version of this Article, N. De Leo and L. Boarino were incorrectly affiliated with ‘Institut de Ciència de Materials de Barcelona (ICMAB–CSIC), Campus de Bellaterra, 08193, Bellaterra, Spain’. The correct affiliation is listed below.

Nanofacility Piemonte INRiM, Nanoscience & Materials Division, Istituto Nazionale di Ricerca Metrologica, Strada delle Cacce 91, 10135, Torino, Italy

This error has now been corrected in the PDF and HTML versions of the Article, and in the accompanying Supplementary Material.

