# Supplementary material for: Impact of pore anisotropy on the thermal conductivity of porous Si nanowires
Source: Sci Rep. 2018 Aug 24;8:12796. doi: 10.1038/s41598-018-30223-0 (PMC6109058; doi:10.1038/s41598-018-30223-0)
Supplement: Supplementary file 1 — Supplementary Information [file 41598_2018_30223_MOESM1_ESM.pdf]

SUPPLEMENTARY INFORMATION

Impact of pore anisotropy on the thermal conductivity of porous Si nanowires

P. Ferrando-Villalba[1], L. D'Ortenzi[2],G. G. Dalkiranis[1], E. Cara[2] A. F. Lopeandia[1], LL. Abad[3], R. Rurali[4], X. Cartoixà[5], N. De Leo[2], Z. Saghi[6], M. Jacob[6], N. Gambacorti[6], L. Boarino[2], J. Rodríguez-Viejo[1,*]

[1]Grup de Nanomaterials i Microsistemes, Departament de Física, Universitat Autònoma de Barcelona, 08193 Bellaterra, Spain.

[2]Nanofacility Piemonte INRiM, Nanoscience & Materials Division, Istituto Nazionale di Ricerca Metrologica, Strada delle Cacce 91 10135 Torino, Italy.

[3]IMB-CNM-CSIC, Campus Bellaterra, 08193 Bellaterra, Spain.

[4]Institut de Ciència de Materials de Barcelona (ICMAB−CSIC), Campus de Bellaterra, 08193 Bellaterra, Spain

[5]Departament d'Enginyeria Electrònica, Universitat Autònoma de Barcelona, 08193 Bellaterra, Spain.

[6]University of Grenoble Alpes, Grenoble F-38000, France; CEA, LETI, MINATEC Campus, Grenoble, F- 38054, France.

FABRICATION

The porous Si nanowires were produced at the Istituto Nazionale di Ricerca Metrologica (INRiM), in Torino, by means of MaCE (Metal-assisted Chemical etching). The main fabrication steps are explained below and depicted in Figure S1.

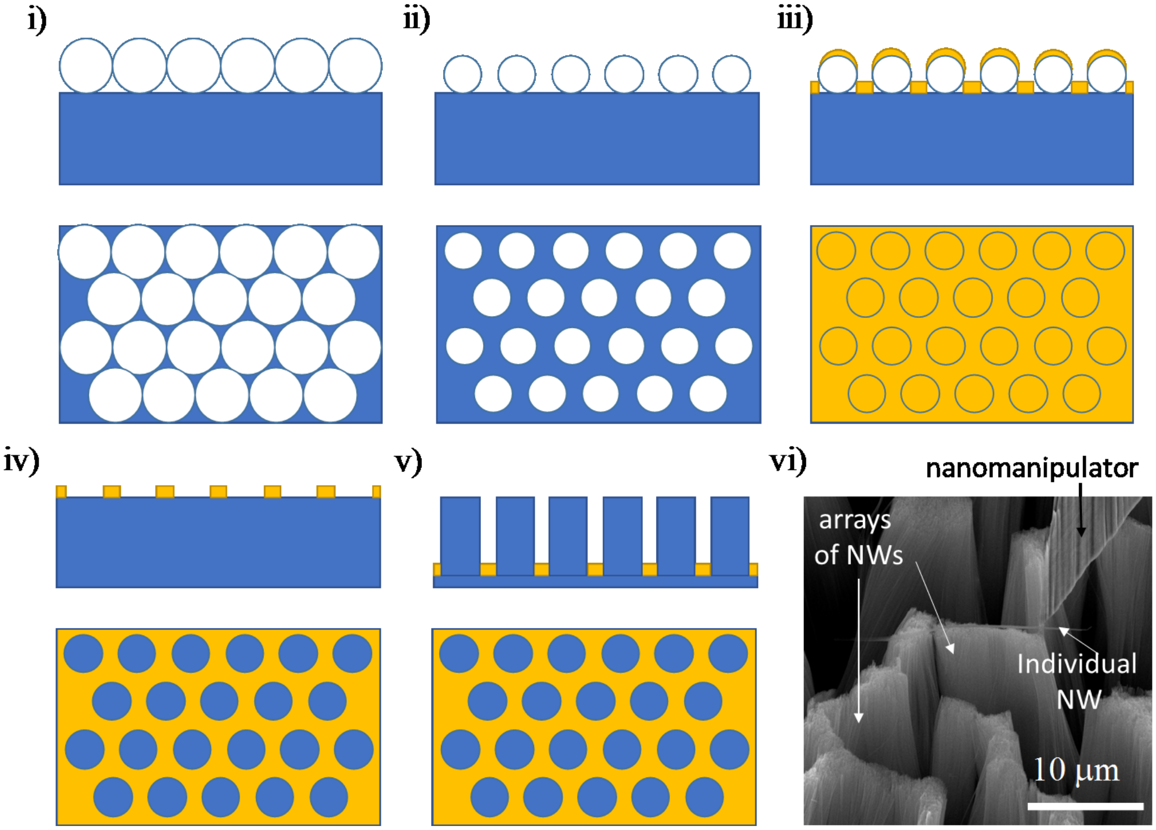

Figure S1 i) to v) Side and top views of each fabrication step. vi) SEM image of 1B nanowires (see Table 1 of the manuscript for the batch reference) showing very long bundled wires as well as the nanomanipulator tip with a wire attached.

i) The process starts with the colloidal lithography, consisting on dispersing on the wafer small polystyrene nanospheres synthesized by emulsion polymerization.

ii) After the first step, the nanospheres are in contact with each other, so they must be reduced in order to produce separated holes in the gold thin-film deposition. This is achieved with an O_2 RIE that selectively etches the organic spheres, effectively reducing their radius.

iii) A 20 nm thick Au layer is deposited by means of e-beam evaporation.

iv) The nanospheres are removed by sonicating the wafer in DI water. The result is a holey Au mask that will be used as a template for the wires to be formed.

v) The nanowires are defined by immersing the wafer into wet etching solutions composed of HF, H_2O_2 and H_2O with proportions of 30:1:30 and 3:1:1. The

etchants concentration and the initial doping level of the wafer define the final porosity of the nanowire.

After the wet etching with HF the Si surface is H-terminated, which passivates the surface against oxidation for a few seconds-to-minutes [1]. However, the combined influence of the electron irradiation inside the SEM and the oxygen from the atmosphere produce the elimination of the H layer and the unavoidable growth of a thin layer of native oxide. The surface passivation of a nanostructure with high surface-to-volume ratio is a very important feature that can alter significantly the value of the thermal conductivity. In particular, a recent molecular dynamics study has shown that the SiO_2 passivation of rough ultrathin Si membranes can reduce the thermal conductivity by up to 80% [2]. For this reason, the very same wires with or without the oxide layer may produce very different results. Testing the influence of surface modifications on the porous nanowires is an interesting avenue of research for future investigations.

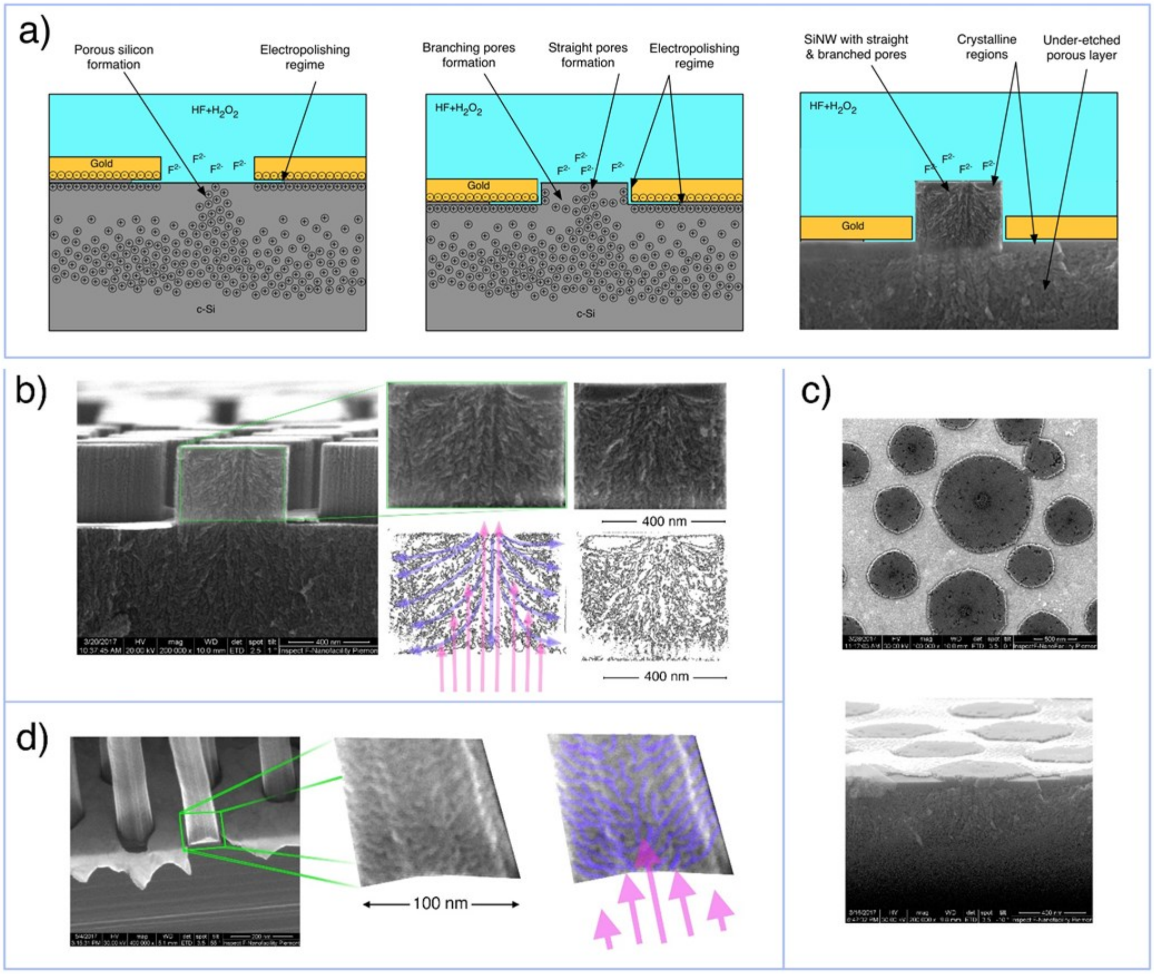

Figure S2. a) Scheme of the formation mechanisms of highly doped Si NWs in the initial moments of etching. b) SEM micrographs of 400–500 nm porous Si NW in cross section, showing the development of porosity into Christmas tree–like structures due to the etching mechanism. c) SEM micrographs of the top view and cross section of the same Si NW after 30 seconds of etching. Unetched crystalline layers clearly appear under the electropolishing zones at the metal-silicon interface and in the top part of the Si NW due to charge depletion. d) Cross-section SEM of a cleaved porous NW that is 100 nm in diameter. The violet lines on the right-hand side figure illustrates the propagation of the pores, in agreement with the mechanism explained in the text. The pink arrows indicate the direction of heat flow during the thermal measurement.

THERMAL CORRECTIONS

Finite Element Modelling:

Finite element modelling (FEM) is necessary to calculate the thermal conductivity of two of the measured wires by subtracting the a-Si thermal resistance of the membrane. A sketch of the simulated structure is shown in S2. The 67 nm Si membrane is considered amorphous up to 2 µm from the edge, so the thermal conductivity of this region is set to 1.2 $W\cdot m^{-1}\cdot K^{-1}$. The hole produced when cutting the wire after placing it has been also modeled as a rectangular void. The wire has been simulated with 2 different lengths, 1.2 and 2 µm and two different widths, 96 nm and 142 nm and with the hole placed 0.1 µm and 0.5 µm away from the contact, respectively.

The simulations performed in DC with the Joule Heating physics package of COMSOL Multiphysics. The total number of polyhedral is approx. 30000.

a) b)

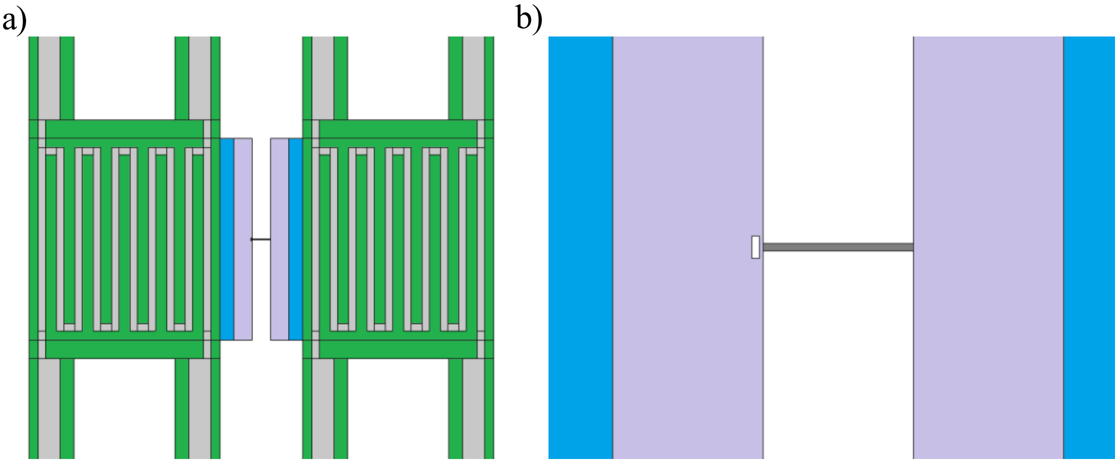

Figure S3: FEM used to find the extra resistance produced by the Si amorphous layer. a) sensing platforms with the partially amorphous Si layer, joined by a wire. b) Zoomed image of the wire, where the hole produced during the wire FIB cutting is appreciated. In this figure, the materials are SiN_x (green), Pt (light gray), c-Si (blue), a-Si (light purple) and porous Si (dark gray).

BACKGROUND SUBTRACTION:

Due to the very low thermal conductance of the nanowires, the background thermal conductance (G_{back}) plays an important role[3]. This conductance is produced by the heat flowing along the beams of the heating bridge, which slightly heats up the substrate increasing the temperature of the sensing platform (S3a). In order to account for this

conductance, a structure with no wire bonding the platforms has been measured in the temperature range of 20 - 320 K (S3b). The conductance of this structure is taken as a reference and it is subsequently subtracted at each temperature from the thermal conductance measured with the nanowires bridging the thermal plates.

We can argue that subtracting the same background conductance from all the measurements is accurate: Firstly, G_{back} is proportional to the thermal conductance from the heaters to the beams (G_{beam}). This has been proven by measuring this value in structures with very different G_{beam}, 83.4 nW/K and 104.9 nW/K, yielding G_{back} values of 0.49 nW/K and 0.62 nW/K respectively. The ratio G_{back}/G_{beam} is 5.9·10^{-3} in both cases, confirming their proportionality.

The structures measured have G_{beam} ranging from 101 nW/K to 109 nW/K. Multiplying this values with the ratio G_{back}/G_{beam} found, we see that this produces G_{back} of 0.6 nW/K and 0.64 nW/K, this is, values of G_{back} within an error of around $\pm$20 pW/K from the value actually used (0.62 nW/K). This error is within the accuracy of the measurement and can only affect the values reported up to 2 % (in the case of the least conductive wire).

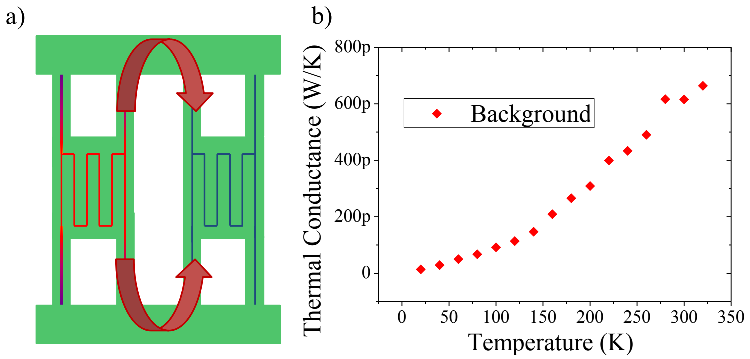

Figure S4 a) Scheme of the heat flow in a structure without sample. b) Background thermal conductance of one of the suspended structures.

References

1. Borini S, Boarino L, Amato G (2006) Anisotropic resistivity of (100)-oriented mesoporous silicon. Appl Phys Lett 89:132111 . doi: 10.1063/1.2357882

2. Neogi S, Reparaz JS, Pereira LFC, et al (2015) Tuning Thermal Transport in Ultrathin Silicon Membranes by Surface Nanoscale Engineering. ACS Nano 9:3820–3828 . doi: 10.1021/nn506792d

3. Ferrando-Villalba P, Lopeandia AF, Abad L, et al (2014) In-plane thermal conductivity of sub-20 nm thick suspended mono-crystalline Si layers. Nanotechnology 25:185402 . doi: 10.1088/0957-4484/25/18/185402
